# Flow-Driven Right-to-Left Shunting Through the Patent Foramen Ovale Causing Hypoxemia and Embolism in a Partial Uhl Anomaly

**DOI:** 10.1016/j.jaccas.2026.108157

**Published:** 2026-05-12

**Authors:** Loreley Eugenia Laboratto, Guido Vannoni, Gabriela Nieto, Julieta Rancati, Lorena Balletti, Germán Cestari, Juan Alberto Gagliardi, Lucía Kazelian, Jorge Lax

**Affiliations:** a Department of Medicine, Division of Cardiology Hospital General de Agudos “Dr. Cosme Argerich” Buenos Aires, Argentina; b Department of Pediatrics, Division of Cardiology Hospital General de Niños “Dr. Pedro de Elizalde” Buenos Aires, Argentina

**Keywords:** bubble echocardiography, cardiac magnetic resonance, congenital heart defect, echocardiography, hypoxemia, right ventricle, right-sided catheterization, stroke, tricuspid valve

## Abstract

**Background:**

Uhl anomaly is an exceptionally rare congenital disorder, with fewer than 100 cases reported. In this setting, a patent foramen ovale may become clinically relevant through non–pressure-dependent hemodynamic mechanisms.

**Case Summary:**

A 49-year-old woman presented with progressive dyspnea and a prior ischemic stroke. Multimodality imaging demonstrated diffuse right ventricular myocardial thinning with global systolic dysfunction and torrential functional tricuspid regurgitation, consistent with partial Uhl anomaly. Despite normal right-sided pressures, a permanent right-to-left shunt through the patent foramen ovale was identified, predominantly driven by flow redirection related to severe tricuspid regurgitation.

**Discussion:**

In rare conditions such as partial Uhl anomaly, integration of multimodality imaging and invasive hemodynamics is essential to establish the diagnosis and to elucidate unconventional mechanisms underlying clinical events.

**Take-Home Messages:**

Partial Uhl anomaly should be considered in adults presenting with unexplained right ventricular dysfunction. Flow-driven right-to-left shunting through a patent foramen ovale may be a major determinant of hypoxemia and embolic risk.

## Visual Summary

**Figure:**
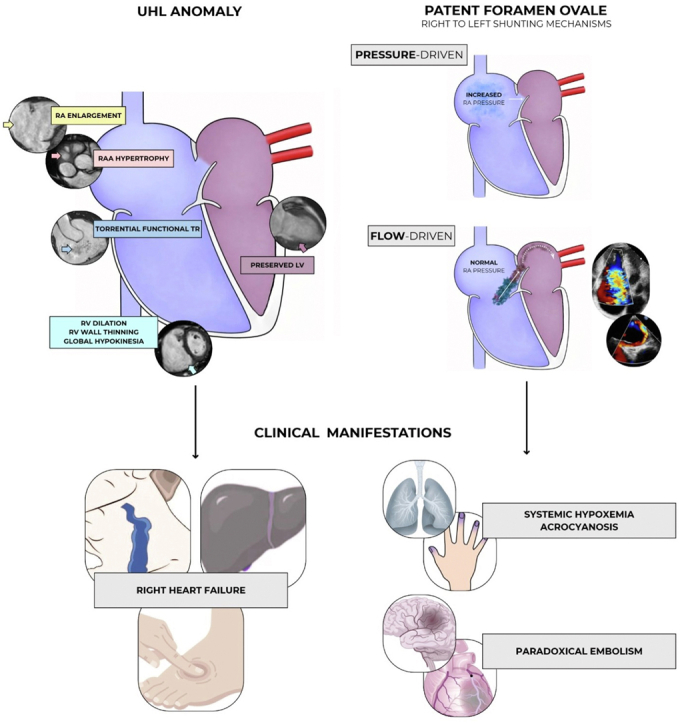

**Visual Summary:**
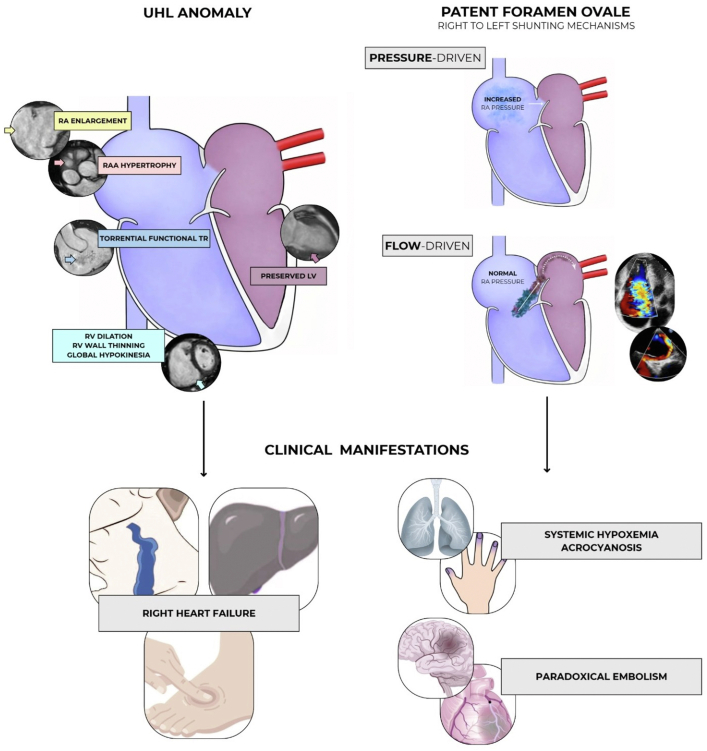
Integrated Pathophysiology of Partial Uhl Anomaly With Patent Foramen Ovale–Mediated Right-to-Left Shunting Structural and functional consequences of partial Uhl anomaly, characterized by right ventricular dilation, wall thinning, global hypokinesia, and severe functional TR with preserved left ventricular function (left). These abnormalities promote right atrial remodeling and facilitate patent foramen ovale–mediated right-to-left shunting through 2 distinct mechanisms: pressure-driven shunting due to elevated right atrial pressure and flow-driven shunting caused, principally, by preferential streaming of the severe TR jet toward the interatrial septum despite normal right atrial pressures (right). The downstream clinical manifestations include right heart failure, systemic hypoxemia with acrocyanosis, and paradoxical embolism. RAA = right atrial appendage; TR = tricuspid regurgitation; other abbreviations as in Figures 4 and 5.

## History of Presentation

A 49-year-old woman presented to the emergency department with several months of NYHA class III dyspnea, with progression to class IV during the week before admission. On arrival, she was normotensive (130/70 mm Hg), tachycardic, and desaturating (82% on room air). Physical examination demonstrated signs of right-sided heart failure, including jugular venous distension, hepatojugular reflux, painful hepatomegaly, and pretibial edema, and acrocyanosis.

**Take-Home Messages**

• Consider partial Uhl anomaly in adults presenting with unexplained RV dilation; multimodality imaging is essential for diagnosis.
• Flow-driven right-to-left shunting through a PFO may result in hypoxemia and embolism, with direct implications for management.

Transthoracic echocardiography revealed severe right atrial (RA) enlargement, right ventricular (RV) dilation with systolic dysfunction, torrential tricuspid regurgitation (TR) secondary to annular dilation, and a patent foramen ovale (PFO). A lung perfusion imaging obtained for suspected pulmonary embolism demonstrated a subsegmental perfusion defect. Oxygen therapy, anticoagulation medication, and loop diuretics were initiated, and the patient was admitted to the coronary care unit for further evaluation and management.

## Past Medical History

The patient had a prior left corticosubcortical parieto-occipital ischemic stroke a year ago without sequelae. Subsequent evaluation included ambulatory monitoring, which documented permanent atrial fibrillation without ventricular ectopy. Over the following months, she developed progressive exertional dyspnea and was awaiting outpatient testing when she experienced symptom worsening, prompting the current admission.

## Differential Diagnosis

The differential diagnosis of RV dysfunction was approached according to the predominant pathophysiological mechanism^1^ (Table 1). Conditions associated with increased RV afterload, including pulmonary hypertension and pulmonary embolism, were considered first. Although imaging findings were consistent with a subsegmental pulmonary embolism, its limited extent could not account for the severity of RV dilation and systolic dysfunction observed on transthoracic echocardiography. Furthermore, and as shown below, right heart catheterization demonstrated normal pulmonary arterial pressures as detailed in the Investigations section.

**Table 1:** RV Dysfunction: Differential Diagnosis

RV pressure overload	RV volume overload	RV cardiomyopathy
Pulmonary hypertension	Valve regurgitation • Tricuspid regurgitation • Pulmonary regurgitation	Myocardial infarction
Pulmonary artery stenosis	System to pulmonary shunt • Interatrial communication • Partial or total anomalous vein drainage	Cardiomyopathies: • Dilated • Hypertrophic • Restrictive • Arrhythmogenic cardiomyopathy of the right ventricle • Amyloidosis • Inflammatory/granulomatous (sarcoidosis) • Other deposits (ie, Danon, Fabry, Pompe)
Pulmonary valve stenosis	Arteriovenous fissure	Myocarditis
Pulmonary embolism	Acute high cardiac output states (ie, thyrotoxicosis)	Ebstein anomaly
		Partial or complete Uhl anomaly
		Sepsis
		Cardiotoxicity (ie, chemotherapy)
		Postcardiotomy syndrome
		Post–left ventricular assist implant

RV = right ventricular.

Etiologies related to RV volume overload were also explored. Severe TR was present; however, multimodality imaging confirmed it to be secondary to annular dilation rather than to primary leaflet pathology. No left-to-right shunt or anomalous venous return was identified. Given the absence of pressure or volume overload sufficient to explain the RV phenotype, a primary RV myocardial disorder was suspected.

The main differential diagnoses considered were Ebstein anomaly and arrhythmogenic RV cardiomyopathy. Normal tricuspid valve morphology and insertion and no apical displacement of the septal leaflet excluded the diagnosis of an Ebstein anomaly. In contrast, arrhythmogenic RV cardiomyopathy was deemed unlikely based on the absence of ventricular arrhythmias on Holter monitoring, lack of depolarization or repolarization abnormalities typically associated with the condition on electrocardiography, no regional RV wall motion abnormalities on cardiac magnetic resonance (CMR) imaging, and no family history suggestive of inherited cardiomyopathy.^2^ On the contrary, CMR findings demonstrated diffuse thinning of the RV wall, predominantly at the inflow tract and inferobasal segments, global RV hypokinesia, reduced RV systolic function, right appendage hypertrophy, and a preserved left ventricle (LV), findings consistent with partial Uhl anomaly^3^ (Table 2).

**Table 2:** Uhl Anomaly vs Arrhythmogenic RV Cardiomyopathy

Feature	Uhl Anomaly	Arrhythmogenic RV Cardiomyopathy
Histopathology	Partial or total absence of RV myocardium	Replacement by fibrofatty tissue
RV wall motion	Global hypokinesia	Regional akinesia or dyskinesia
Ventricular arrhythmias	Not associated	Characteristic
Risk of sudden death	Low	High
Family history/genetics	Absent	Present
Age at presentation	Infancy (except partial forms)	Adolescence/adulthood
Typical mode of presentation	Right-sided heart failure	Ventricular arrhythmias, syncope, or sudden death
Associated genetic mutation	Not identified	Desmosomal genes (ie, desmoplakin, plakophilin, etc)
Electrocardiogram	Nonspecific	Specific depolarization and repolarization abnormalities (epsilon waves, negative T waves in right precordial leads, etc)

Abbreviation as in Table 1.

## Investigations

Electrocardiography demonstrated atrial fibrillation with nonspecific repolarization alterations (Figure 1). Chest radiography revealed cardiomegaly at the expense of the right-sided chambers (Figure 2), without evidence of pulmonary venous redistribution. A lung perfusion imaging showed a subsegmental perfusion defect (Figure 3).

**Figure 1:**
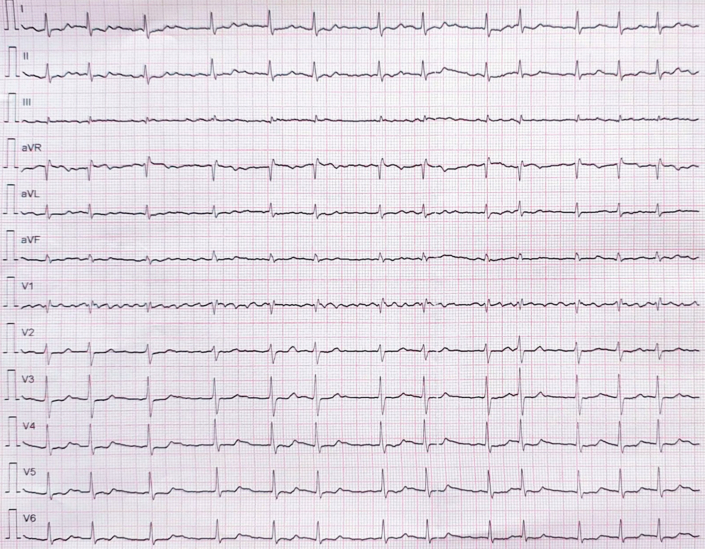
Electrocardiogram Atrial fibrillation as seen with an adequate ventricular response. Nonspecific repolarization alterations are also seen.

**Figure 2:**
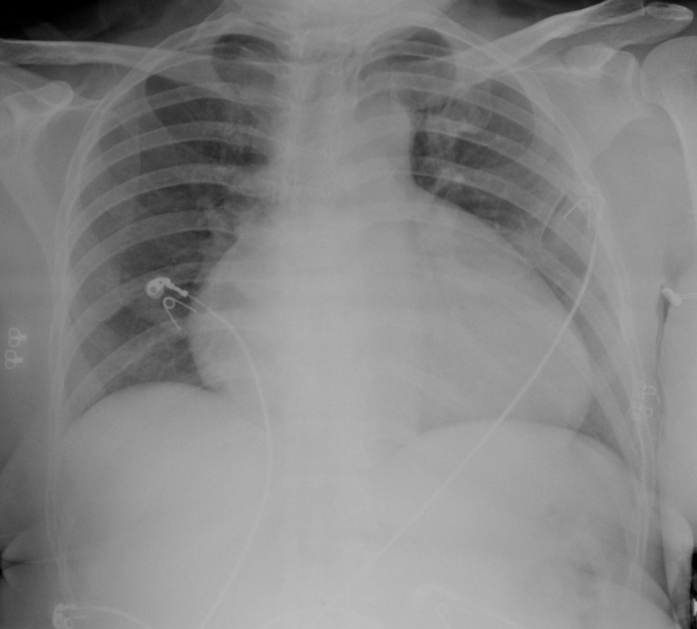
Chest Radiography Cardiomegaly at the expense of right-sided chambers.

**Figure 3:**
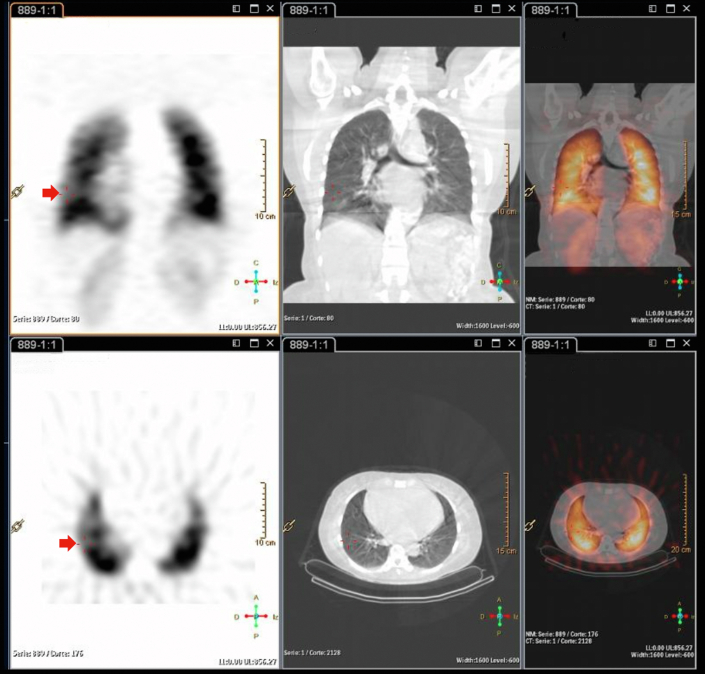
Lung Perfusion Imaging With Hybrid Single-Proton Emission Computed Tomography/Computed Tomography Hybrid single-proton emission computed tomography/computed tomography lung perfusion imaging demonstrating a small peripheral subsegmental perfusion defect (red arrows), consistent with low-burden pulmonary embolism.

Transthoracic echocardiography revealed a nondilated LV with preserved systolic function and no regional wall-motion abnormalities, along with a markedly dilated right ventricle with impaired systolic function and pronounced RA enlargement. Torrential functional TR due to annular dilation was also observed, producing an eccentric regurgitant jet directed toward the interatrial septum (Video 1). On transesophageal echocardiography, the regurgitant jet was further characterized, demonstrating extension into the left atrium with a prominent Coandă effect, consistent with a PFO-mediated shunt (Video 2). Agitated saline contrast demonstrated massive early right-to-left bubble passage across the PFO, without positional differences between the supine and upright states (Video 3, Figure 4).

**Figure 4:**
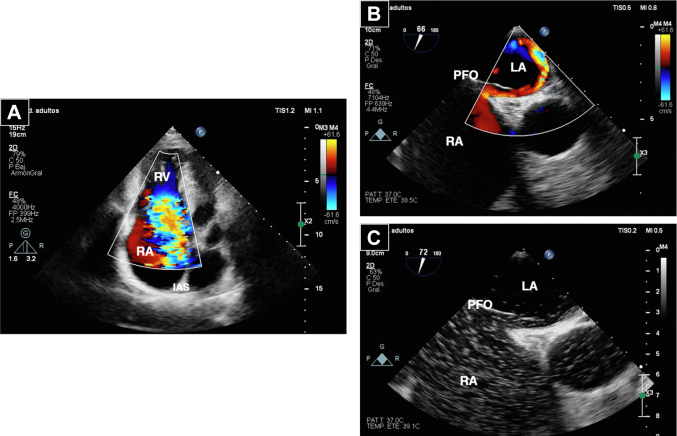
Echocardiography Transthoracic echocardiography in the apical 4-chamber view shows right-sided chamber dilation and torrential functional tricuspid regurgitation due to annular dilatation, with the regurgitant jet directed toward the interatrial septum (A). Transesophageal echocardiography demonstrates a PFO with right-to-left shunt and a torrential tricuspid regurgitation jet crossing the LA with a Coandă effect (B), along with massive right-to-left microbubble passage after agitated saline injection (C). IAS = interatrial septum; LA = left atrium; PFO = patent foramen ovale; RA = right atrium; RV = right ventricle.

CMR imaging showed a moderately dilated right ventricle, with diffuse thinning of its wall, predominantly at the inflow tract and inferobasal segments; global hypokinesia and moderately reduced systolic function; severe RA enlargement; and right appendage hypertrophy. The LV exhibited preserved systolic function and absence of segmental abnormalities. A predominantly right-to-left interatrial shunt was confirmed, with a Q_p_/Q_s_ ratio of 0.9. Late gadolinium enhancement included a linear pattern along the inferior inflow tract of the right ventricle, corresponding to regions of minimal myocardial thickness; a small focal inferolateral basal scar consistent with a probable embolic infarct in the LV; and, although the pericardium was not thickened and there was no pericardial effusion, mild circumferential late gadolinium enhancement along the pericardium, consistent with residual changes from prior pericardial inflammation. Importantly, there were no imaging or hemodynamic features of constrictive physiology, and this finding was considered incidental and unrelated to the patient's clinical and hemodynamic presentation. CMR images were analyzed using the freely available software Segment 4.0.0.1 (Medviso) (Figure 5, Video 4).

**Figure 5:**
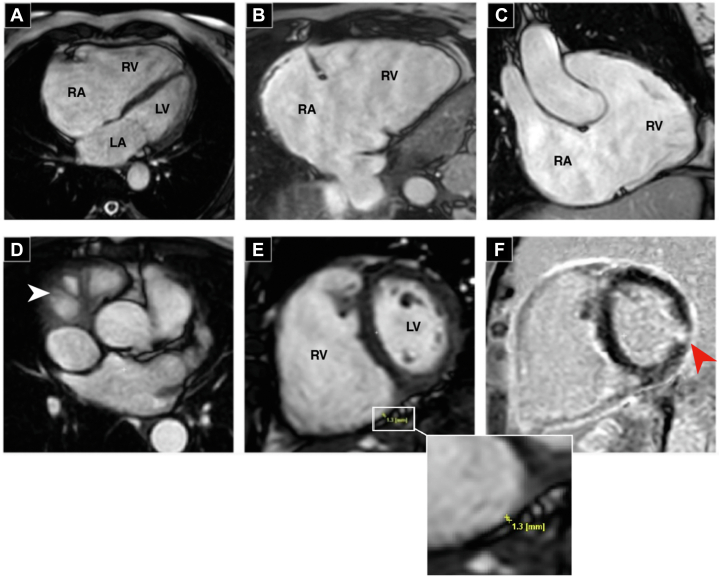
Cardiac Magnetic Resonance Imaging Cardiac magnetic resonance cine images in long- and short-axis views demonstrate a moderately dilated RV with diffuse wall thinning, predominantly involving the inflow tract and inferobasal segments, with a minimum measured wall thickness of 1.3 mm (A-C and E). Marked right atrial dilation is present (A-C), with a hypertrophied right atrial appendage (D, white arrow). The LV remains of normal size (A and E). Late gadolinium enhancement images (F) demonstrate a linear enhancement pattern along the inferior right ventricular inflow tract, corresponding to regions of minimal myocardial thickness; a small focal inferolateral basal left ventricular scar, consistent with a probable embolic infarction (red arrow); and mild circumferential pericardial late gadolinium enhancement without pericardial thickening or effusion, compatible with residual changes from prior pericardial inflammation. LV = left ventricle; other abbreviations as in Figure 4.

A right heart catheterization, performed once the patient had achieved an optimal volume-depleted state, revealed normal right- and left-sided filling pressures, low pulmonary vascular resistance, a preserved cardiac index, and a net right-to-left shunt (Table 3). These integrated findings support a diagnosis of partial Uhl anomaly with torrential TR and permanent right-to-left shunting through a PFO, driven by mixed pressure- and flow-related physiology, resulting in systemic hypoxemia and paradoxical embolism and guiding the proposed therapeutic approach.

**Table 3:** Right Heart Catheterization Measurements

Hemodynamic Parameters	Obtained Values
Pulmonary artery, mm Hg	19/14 (16)
Pulmonary capillary wedge pressure, mm Hg	14
Right atrium, mm Hg	5
Cardiac output, L/min	5.2
Pulmonary vascular resistance, Wood units	0.37
Q_p_/Q_s_ ratio	0.67

## Management

Given the presence of a PFO with clinically significant consequences, including paradoxical embolism and persistent hypoxemia, a comprehensive assessment of the risks and benefits of PFO closure is warranted. Current American Heart Association/American Stroke Association guidance supports percutaneous PFO closure in patients aged 18 to 60 years with a nonlacunar ischemic stroke of undetermined cause and a PFO with high-risk anatomical features.^4^

In this patient, multiple features strongly suggest a causal role of the PFO: embolic infarcts in 2 territories, coexisting pulmonary embolism, and the magnitude of the right-to-left shunt. These findings alone justify closure for secondary embolic prevention. Furthermore, the patient exhibited profound hypoxemia attributable to right-to-left shunting across the PFO in the absence of any other condition that could explain her oxygenation defect. In such scenarios, percutaneous closure has demonstrated marked and rapid improvement in systemic oxygenation, with a favorable safety profile.^5^

Although percutaneous PFO closure was initially considered, the patient's complex anatomy and physiology prompted a consensus surgical strategy after multidisciplinary heart team evaluation. This approach enables simultaneous PFO closure and tricuspid valve repair, addressing both the interatrial conduit for right-to-left shunting and its primary hemodynamic driver.

Nevertheless, it should be considered that PFO may also function as a partially compensatory decompressive pathway in advanced RV disease. Abrupt elimination of this shunt therefore carries a risk of precipitating acute RV failure. Accordingly, temporary balloon occlusion of the PFO was planned before definitive surgical intervention to assess hemodynamic tolerance, including changes in right-sided filling pressures, cardiac output, and systemic oxygenation.

## Discussion

Uhl anomaly is characterized by partial or complete absence of the RV myocardium,^6^ believed to result from dysregulated myocyte apoptosis.^7^ This structural deficiency leads to progressive RV dilation, global systolic impairment, and markedly RA dilation with right heart failure. Although most affected patients present at a young age, adult-onset forms have been described, typically manifesting as progressive right-sided failure.^8^ CMR imaging is the diagnostic modality of choice,^3^ and, in our patient, multimodality imaging demonstrated the hallmarks of partial Uhl anomaly.

PFO has been reported in patients with Uhl anomaly, with a prevalence similar to that of the general population. Although most PFOs remain clinically silent, they may serve as conduits for paradoxical embolism, systemic hypoxemia, and even platypnea-orthodeoxia syndrome.

Hypoxemia classically occurs through a pressure-driven right-to-left shunt, in which elevated RA pressure forces desaturated blood across the interatrial septum. However, a flow-driven mechanism may also occur—even with normal right-sided pressures—when geometric distortion of the interatrial septum or directional blood flow preferentially directs venous return toward the PFO.^9^

In this patient, severe TR played a pivotal role in perpetuating the shunt. The regurgitant jet was directed toward the interatrial septum, producing vectorial redirection of flow across the PFO, thereby sustaining a flow-driven right-to-left shunt despite normal intracardiac pressures after decongestion. RA dilation, exacerbated by chronic volume overload from TR and longstanding atrial fibrillation, further facilitated septal displacement and maintained channel patency. Although oxygenation improved with decongestion, reflecting partial reduction of the pressure component, hypoxemia persisted because of the dominant flow-driven mechanism. This pathophysiology also contextualizes her prior paradoxical embolic stroke.

Understanding these interacting structural and hemodynamic factors is crucial because they inform therapeutic decision-making. In adult patients with partial Uhl anomaly, management options are limited, and heart transplantation remains the definitive treatment for advanced disease. In the interim, targeted management of TR and PFO may palliate symptoms and reduce embolic risk.

## Follow-Up

The patient is currently managed with anticoagulation and diuretic medications while awaiting planned surgical PFO closure and tricuspid valve repair. The team will reassess hemodynamic tolerance during temporary balloon occlusion before definitive intervention. Long-term follow-up will include surveillance for recurrent embolic events, progression of right heart dysfunction, and evaluation for heart transplantation candidacy if symptoms or RV function deteriorate.

## Conclusions

Partial Uhl anomaly is a rare cause of right-sided heart failure. In this patient, a permanent right-to-left PFO shunt, due to a mixed pressure- and flow-driven physiology, led to systemic hypoxemia and paradoxical embolism. Recognition of these interacting pathophysiological factors is crucial for targeted interventions, including PFO closure and tricuspid repair, whereas heart transplantation remains the definitive therapy in advanced disease.

## Funding Support and Author Disclosures

The authors have reported that they have no relationships relevant to the contents of this paper to disclose.

## Appendix

Video 1 Transthoracic Echocardiography, Apical 4-Chamber View (Color Doppler). Apical 4-chamber color Doppler echocardiographic view showing right-sided chamber dilation and torrential functional tricuspid regurgitation due to annular dilatation, with the regurgitant jet directed toward the interatrial septum.

Video 2 Transesophageal Echocardiography With Color Doppler. Transesophageal echocardiography with color Doppler demonstrating a patent foramen ovale with right-to-left shunting and a torrential tricuspid regurgitation jet crossing the left atrium with a Coandă effect.

Video 3 Transesophageal Echocardiography With Agitated Saline Contrast. Transesophageal echocardiography with agitated saline contrast showing a massive right-to-left microbubble passage through the patent foramen ovale.

Video 4 Cardiac Magnetic Resonance, Axial Cine View. Cardiac magnetic resonance axial cine sequence showing moderate right ventricular dilation with diffuse wall thinning and severe right atrial enlargement, along with torrential functional tricuspid regurgitation due to annular dilatation. Right ventricular systolic function is moderately reduced, with no regional wall motion abnormalities of the free wall.
